# Enhancement of Neurite Outgrowth by Warming Biomaterial Ultrasound Treatment

**DOI:** 10.3390/ijms21062236

**Published:** 2020-03-23

**Authors:** Jung-Chih Chen, Chao-Ming Su, Gin-Shin Chen, Chin-Chun Lai, Ching-Yun Chen, Kurt Ming-Chao Lin, Feng-Huei Lin, Guo-Chung Dong

**Affiliations:** 1 Institute of Biomedical Engineering, National Chiao Tung University, Hsinchu City 300-41, Taiwan; george@nctu.edu.tw; 2 Institute of Biomedical Engineering and Nanomedicine, National Health Research Institutes, Miaoli County 358-69, Taiwan; CMSU@nhri.org.tw (C.-M.S.); gschen@nhri.edu.tw (G.-S.C.); amberlai.cm08g@nctu.edu.tw (C.-C.L.); chingyun523@gmail.com (C.-Y.C.); Klin@nhri.edu.tw (K.M.-C.L.)

**Keywords:** ultrasound, thermal effect, neurite outgrowth, sonosensitized biomaterial, acoustic attenuation

## Abstract

Ultrasound is a method for enhancing neurite outgrowth because of its thermal effect. In order to reach the working temperature to enhance neurite outgrowth, long-time treatment by ultrasound is necessary, while acknowledging that the treatment poses a high risk of damaging nerve cells. To overcome this problem, we developed a method that shortens the ultrasonic treatment time with a warming biomaterial. In this study, we used Fe_3_O_4_ nanoparticle-embedded polycaprolactone (PCL) as a sonosensitized biomaterial, which has an excellent heating rate due to its high acoustic attenuation. With this material, the ultrasonic treatment time for enhancing neurite outgrowth could be effectively shortened. Ultrasonic treatment could also increase neuronal function combined with the warming biomaterial, with more promoter neuronal function than only ultrasound. Moreover, the risk of overexposure can be avoided by the use of the warming biomaterial by reducing the ultrasonic treatment time, providing better effectiveness.

## 1. Introduction

Peripheral nerve injuries are very common, in which nerve innervation is affected and can lead to a loss of function, including nerve-to-nerve or nerve-to-organ connections [1]. The use of nerve conduits is a clinical treatment for nerve damage by promoting axonal regeneration [2,3,4] and avoiding misalignment during correction [5]. However, nerve conduit treatment can take more than a year of recovery time [2]. Physiotherapists have been using therapeutic ultrasound or thermal therapy mostly to repair soft tissue injuries, such as damage to muscles or nerves [6,7,8]. Thermal stimulation of neural cells at 39.5–40 °C [9] can enhance neurite outgrowth, but therapeutic ultrasound can also induce nerve damage or cause local tissue injury due to the thermal effect of cavitation. Attenuation is determined by ultrasound frequency and tissue depth [10,11,12,13]. As ultrasound travels through the surface and into the tissue, attenuation decreases, which makes it difficult to achieve the required treatment temperature for deeper damaged tissues.

Magnetic nanoparticles have been used as sonosensitizing materials for ultrasound-induced thermal treatment [13,14,15,16] owing to their high attenuation. The effect of the heating rate was enhanced in magnetic nanoparticle containing-biomaterials by using an ultrasound system [17]. Polycaprolactone (PCL) has been approved by the Food and Drug Administration (FDA) and Conformit Europe (CE) for use in nerve conduit applications [2]. For this study, we used magnetic nanoparticles Fe_3_O_4_ as a sonosensitizing material to enhance the thermal effect of ultrasonic treatment for neurite outgrowth. Moreover, Fe_3_O_4_ nanoparticles were embedded into a PCL membrane as a warming biomaterial to enhance neurite outgrowth by ultrasonic treatment of neural cells.

## 2. Results

### 2.1. Properties of Warming Biomaterial

**Figure 1:**
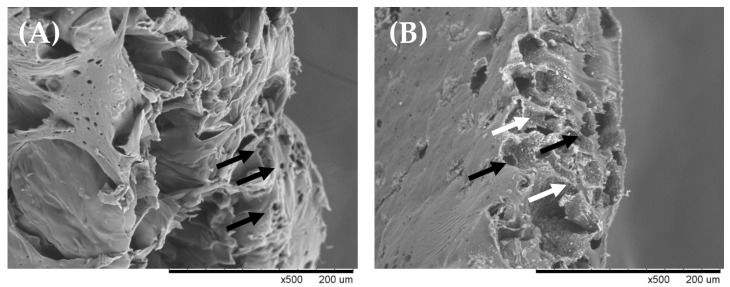
Morphology of biomaterials longitudinal sections by scanning electron microscope. (**A**) PCL membrane, and (**B**) warming biomaterial. White arrows indicate magnetic nanoparticles and black arrows indicate pores of biomaterials.

**Figure 2:**
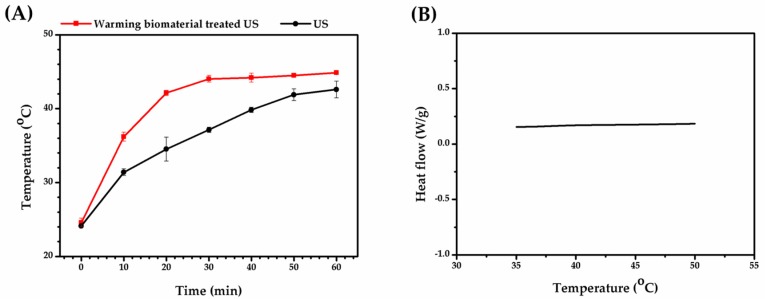
Warming biomaterial properties. (**A**) Temperature curve of warming biomaterial treated with ultrasound (US). (**B**) Differential scanning colorimetry (DSC) result of warming biomaterial.

Fe_3_O_4_ nanoparticles embedded in PCL were used as a sonosensitizing warming biomaterial due to their high attenuation. High attenuation means more energy is absorbed by the warming biomaterial during treatment. As shown in Table 1, the attenuation of the warming biomaterial was 739.99 ± 3.56 dB/cm·MHz, which has a higher sonosensitizing property than PCL without Fe_3_O_4_ (100.84 ± 0.87 dB/cm·MHz). In Figure 1A, it is shown that the membrane of PCL materials had porosity and the pores were irregular in their distribution. In Figure 1B, the magnetic nanoparticles were uniformly distributed on warming biomaterial so that the warming biomaterial could uniformly heat by ultrasonic treatment. The thermal conductivity of PCL was low (~0.3 W/m·k) [18], so the particle distribution was an important factor in the therapeutic effect. With this characteristic, cells withstand uniform thermal treatment to improve neurite growth consistency. As shown in Figure 2A, we measured the temperature of the ultrasound-induced warmed biomaterial. When water was treated with ultrasound, the temperature reached 40 °C after 41 min, which is the required temperature for enhancing neurite outgrowth. On the other hand, the temperature curve of the warming biomaterial showed a high heating rate, indicating that the projected temperature could be achieved after only 16 min of ultrasonic treatment. This denotes that the warming biomaterial could reduce the extent of ultrasound treatment, avoiding overstimulation. To check the thermal stability of the biomaterial, differential scanning calorimetry (DSC) was done. As shown in Figure 2B, the warmed biomaterial did not display any obvious endothermic/exothermic peak from 35–50 °C, indicating that it was indeed stable when treated with ultrasound. In our strategy, the warming biomaterial would be implanted into a nerve damage site and heated by ultrasound; hence, the thermal stability of the warmed biomaterial is very important for future treatments.

### 2.2. Cell Viability of Warming Biomaterial with Ultrasound Combined Treatment

**Figure 3:**
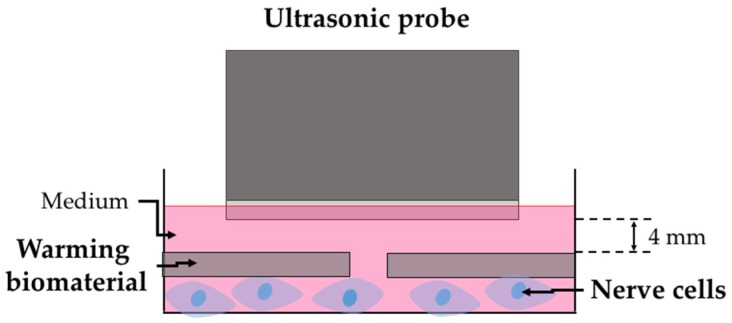
Schematic diagram of warming biomaterial combined with ultrasound on nerve cells.

**Figure 4:**
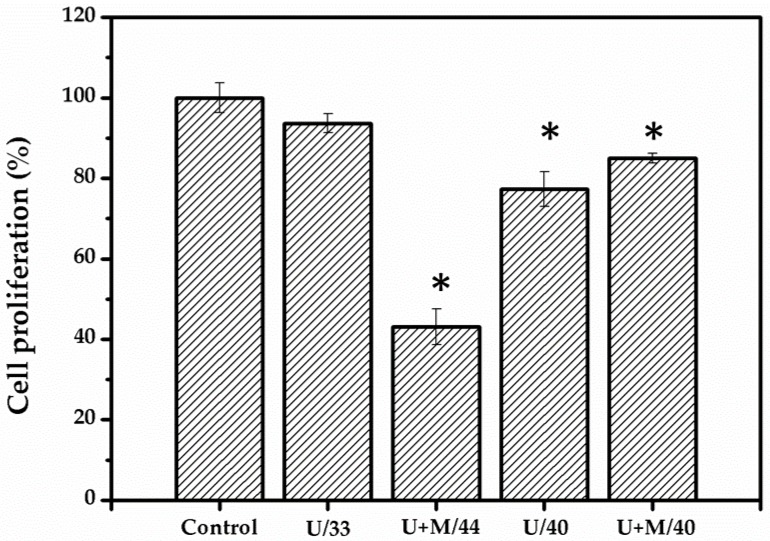
Cell proliferation results of ultrasound-treated N2A cells. Nontreated cells are designated as the control, U/33 is ultrasound-treated cells at 33 °C, U+M/44 is ultrasound-treated cells combined with warming biomaterial at 44 °C, U/40 is ultrasound-treated cells at 40 °C, and U+M/40 is ultrasound-treated cells combined with warming biomaterial at 40 °C.

Ultrasound was combined with the warming biomaterial for treatment, as shown in Figure 3. N2A cells were treated with ultrasound for 16 min and retained their viability at 93%. However, the temperature was only 33 °C, which does not reach the required temperature for neurite outgrowth. By prolonging the treatment duration to 43 min (40 °C), the cell viability decreased to 77%. Warming biomaterial was used to cover N2A cells prior to ultrasound treatment (Figure 4). The temperature was increased to 40 °C after 16 min of treatment. The cell viability was 85%, which is slightly higher than with the ultrasound treatment alone at 40 °C. The exposure period was then continued up to 43 min, and the cell viability decreased to 43% due to overheating (44 °C), which damaged the cells.

### 2.3. Warming Biomaterial with Ultrasound Combined Treatment Improves Nerve Outgrowth

**Figure 5:**
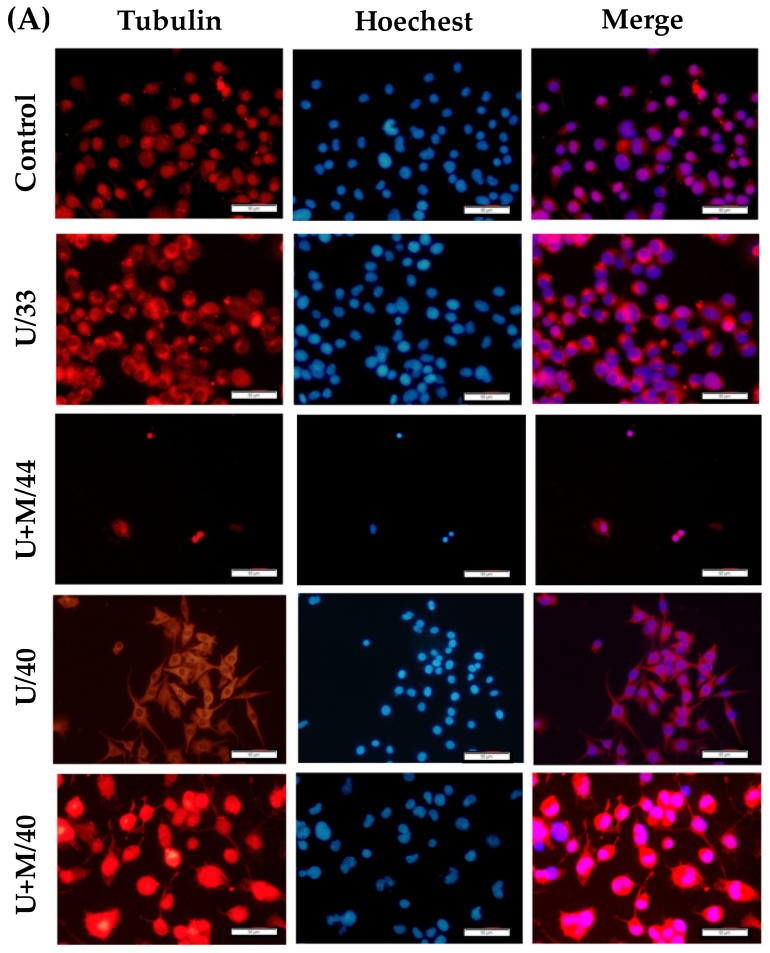

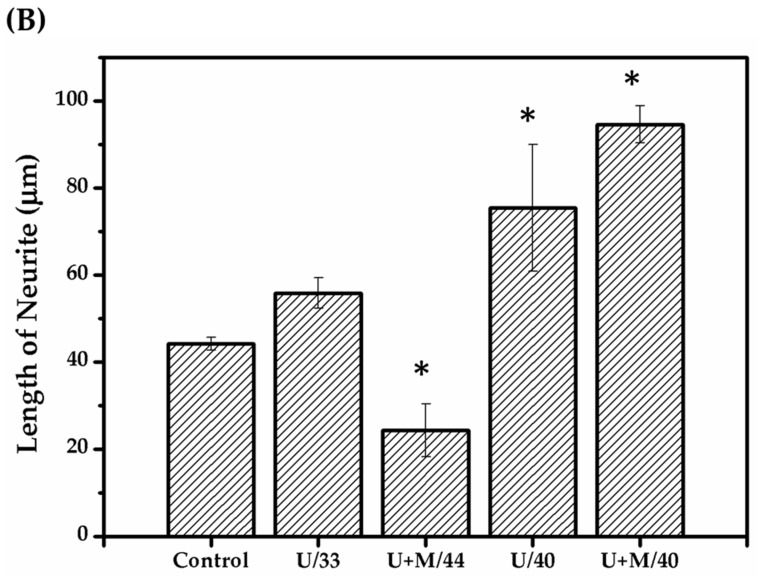
Fluorescence staining and nerve outgrowth analysis. (**A**) Fluorescent images of ultrasound-treated cells combined with the warming biomaterial to enhance nerve outgrowth. Non-treated cells are designated as the control, U/33 is ultrasound-treated cells at 33 °C, U+M/44 is ultrasound-treated cells combined with warming biomaterial at 44 °C, U/40 is ultrasound-treated cells at 40 °C, U+M/40 is ultrasound-treated cells combined with warming biomaterial at 40 °C. Scale bar = 50 μm. (**B**) Quantification of neurite length by fluorescent image. * *p* < 0.05 compared with control group.

As seen from the fluorescent images in Figure 5A, nerve cells from the control and U/33 groups did not show any obvious axonal growth but displayed an increase in the length of outgrowth when treated with ultrasound at 40 °C (U/40). A similar phenomenon was also seen when warming biomaterial was combined with ultrasound treatment. The warming biomaterial was heated to 40 °C after 16 min of ultrasonic treatment, thereby inducing nerve outgrowth. The length of the nerve outgrowth when comparing the U/40 and control groups showed significant improvement. Figure 5B shows the quantitative analysis of nerve outgrowth from fluorescent images. The warming biomaterial combined with ultrasonic treatment induced longer axonal length than the ultrasound treatment alone. This was due to the effect of the exposure period on nerve outgrowth.

### 2.4. Warming Biomaterial with Ultrasound Combined Treatment Enhances AChE Activity

**Figure 6:**
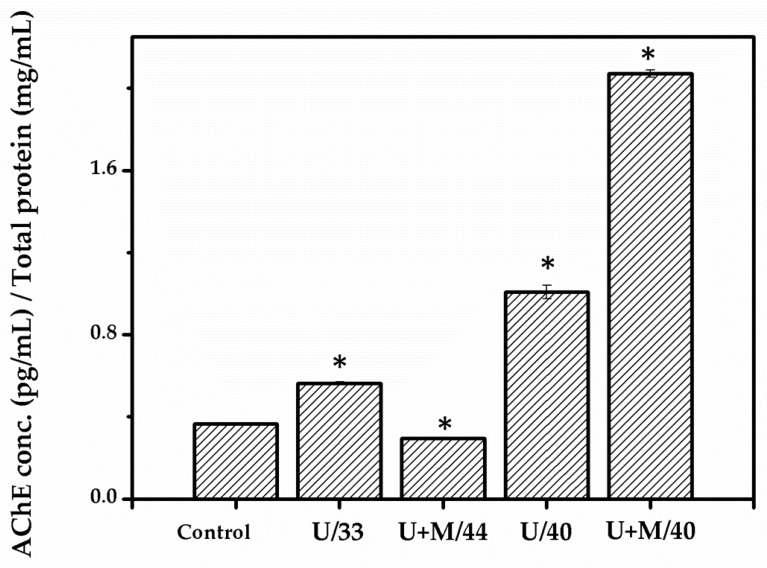
Measurement of acetylcholinesterase (AChE) activity. Non-treated cells are designated as control, U/33 is ultrasound-treated cells at 33 °C, U+M/44 is ultrasound-treated cells combined with warming biomaterial at 44 °C, U/40 is ultrasound-treated cells at 40 °C, U+M/40 is ultrasound-treated cells combined with warming biomaterial at 40 °C. * *p* < 0.05 compared with control group.

Acetylcholinesterase (AChE) activity is a biomarker for neuronal function. We assessed whether the warming biomaterial, when combined with ultrasound, could increase the AChE activity of cells. As shown in Figure 6, AChE activity slightly improved at 33 °C of ultrasonic treatment. However, at U+M/44 °C, AChE activity was weak due to nerve cell damage by overheating. In contrast to 40 °C treatment, AChE activity significantly increased with either ultrasound alone or in combination with the warming biomaterial.

## 3. Discussion

Ultrasound-induced heating through ultrasonic wave attenuation and conversion of its energy into heat was examined. The effectiveness of ultrasonic-induced heating could be significantly improved by a sonosensitizer, such as magnetic nanoparticles embedded in warming biomaterial [14,19]. Ultrasonic treated particles induced the crystal lattice change had been reported [20], but we still cannot confirm the heating of warming biomaterials treated with ultrasound due to this cause. However, we still suggest some possible reasons for ultrasound-induced heating. Nanoparticle heating by ultrasound treatment had three mechanisms, and differences in compressibility, the thermal transport process, the density between the dispersed particles, and in the continuous phase. Magnetic particles were embedded in warming biomaterials, so that they lacked the interaction of differences in compressibility, and the particles also had poor thermal transport effectivity, so the nanoparticles of the warming biomaterial heating mechanism were related in high density of magnetic nanoparticles to induce thermal effect [17]. In Józefczak’s study [21], the attenuation of increased sonosensitizer enhanced ultrasonic-induced heating effectiveness, and the employed sonosensitizing material could obviously promote the heating rate compared to tissue-mimicking phantoms. Magnetic particles were embedded on warming biomaterials, so that there were no interaction differences in compressibility. The warming biomaterial had a high attenuation of about 740 dB/cm·MHz. This means that most of the ultrasound energy was absorbed in the biomaterial, converting it to heat. The attenuation of tissues such as muscle, skin, fat, and bone was 1.1–4.1 [22], 0.8–3.6 [23], 0.08–0.39 [11], and 13.3 [24] dB/cm·MHz, respectively. The attenuation of warming biomaterial shows great differences from normal tissue, so ultrasound exposure of warming biomaterial implanted tissue would cause the heating rate to increase more dramatically than the surrounding tissue. This strategy shows that different temperatures in specific locations achieve thermal-induced neurite outgrowth and reduce the risk of overheating surrounding tissue.

This signifies that when the biomaterial is implanted in the target site and treated with ultrasound, only specific locations are heated, leaving the temperature of surrounding tissues unchanged. In this case, the treatment time can be reduced to avoid the risk of damage by overtreatment.

Another advantage is that the biomaterial has high sensitivity, which means there is a high possibility for inducing heat when implanted in deeper tissues in the body. As shown in Figure 4, the U+W/44 group overheated due to a long treatment time (43 min), but the duration was the same as that of the U/40 group. This implies that the warming biomaterial can be used effectively by reducing the ultrasound treatment time. The decreased cell numbers in the U+M/40 and U/40 groups may have been induced by lower proliferative activity compared with the control group. This can be of benefit to increase neurite outgrowth. In addition, the decrease in the U+M/40 group was more than in the U/40 group. The higher neurite outgrowth in the U+M/40 group is also confirmed in Figure 4.

Thermally stimulated cells are confirmed to enhance neurite outgrowth through activation and signaling of the p38 mitogen-activated protein kinase (MAPK) pathway [9,25,26] in PC12 cells, and there is a lack of relevant studies on N2A cells but some studies have explored the MAPK pathway related to neurite outgrowth in N2A cells [27,28]. Heat stimulation activates phosphatidylinositol 3-kinase (PI3K)/Akt and the three MAPK signal transduction pathways. In our study, heat was generated in the warming biomaterial through exposure to ultrasound. The enhancement of neurite outgrowth was possibly due to the activation of the p38 MAPK pathway from the thermally stimulated cells. Through the ultrasonic treatment of the warming biomaterial with N2A cells, neurite outgrowth was induced, extending the axonal length by about twice that of the control group (Figure 5). This result was similar to Kudo’s study [9], which confirmed that the mechanism of neurite outgrowth was mainly due to heat induction; however, our AChE activity results are higher compared to theirs. This can be attributed to the shrinkage of the membrane due to acoustic overstimulation affecting the distribution of acetylcholinesterase, thereby promoting neurotransmission from the presynaptic to the postsynaptic neurons. In Hu’s results, the neuronal cells gradually withdrew one or more of their neurites over the exposure period. These results show that pulsed ultrasound is capable of instigating repulsive changes in neuronal morphology in vitro [29]. As shown in Figure 5B, the cell shape was slenderer when exposed to ultrasonic treatment in the U/40 group than in U+M/40 group. This is possibly due to ultrasonic treatment inducing cells to retract to a significantly smaller area. In the U+M/40 group, almost all ultrasonic power was converted to a thermal effect that avoided ultrasound-induced retraction [29], so the morphology of the cells of the U+M/40 group shows that they were bigger and more swollen than in the U/40 group.

However, the mechanism of neurite outgrowth by both a thermal and an acoustic effect has yet to be defined. In the present study, we demonstrated that the developed biomaterial has the property of high ultrasonic absorption compared to human soft tissues. For clinical applications, the distance between the ultrasound head and the biomaterial/target nerve is several centimeters, and higher ultrasound power is required to heat the biomaterial due to the attenuation in the medium tissues such as skin, fat, and muscle. When the biomaterial reaches the desired temperature, we infer that the temperature of the medium tissues is still low because of the considerable difference in ultrasonic absorption between the biomaterial and the medium tissues. Further study is needed.

In this study, we used a low-power homemade ultrasound device that can generate heat in the warming biomaterial. This implies that the biomaterial can be used in a clinical setting without the need for expensive equipment. In addition, FDA-approved nerve conduits are currently limited to <3 cm of nerve defects, making it hard to track the efficacy of treatment. The warming biomaterial is ultrasound-sensitive. By implanting the biomaterial as a nerve conduit for severed nerves, the exact treatment location of a nerve injury can be visualized while doing the treatment at the same time.

## 4. Materials and Methods

### 4.1. Chemicals and Reagents

Gelatin, polycaprolactone, WST-1 cell proliferation reagent, and formaldehyde were purchased from Sigma-Aldrich Inc. (St. Louis, MO, USA). Hoechst 33341 was purchased from Abcam (Cambridge, MA, USA). Alexa Fluor^®^ 594 anti-tubulin beta-3 was purchased from BioLegend (San Diego, CA, USA). Dulbecco’s Modified Eagle Medium, penicillin/streptomycin solution, trypsin, and fetal bovine serum were from Thermo Fisher Scientific (Waltham, MA, USA).

### 4.2. Warming Biomaterial Preparation

PCL and Fe_3_O_4_ nanoparticles were dissolved in dichloromethane and a solution was prepared that contained 7.5% PCL/3% Fe_3_O_4_. Then 15 mL of PCL/Fe_3_O_4_ solution was mixed with 10% gelatin solution in a 9:1 ratio. Fe_3_O_4_ nanoparticles from the mixed solution were then dispersed via sonication for 1 h. The mixed solution was poured into a glass dish (diameter: 10 cm) and dried in a fume hood overnight. The formed membrane was washed several times with distilled water and trimmed to a round shape with a diameter of 3.5 cm.

### 4.3. Microscopic Observation

Microscopic observation was carried out by scanning electron microscope (SEM; TM1000, Hitachi) and the magnetic nanoparticle distribution and morphology of warming biomaterial were observed. The sample was coated with Au, and SEM observation was with an operating voltage of 15 kV and the vacuum level of the observation chamber was 10^–5^ to 10^–7^ Pa.

### 4.4. Measurement of Ultrasonic Characteristics of Warming Biomaterial

To measure the attenuation of the warming membrane and for other succeeding experiments, a homemade ultrasonic probe was used. The probe is connected to a pulser (Olympus, 5072 pr pulser/receiver) to generate pulses (1.025 MHz) on warming biomaterial with a 4 mm distance in-between. Then, the voltage of the acoustic field was measured using a hydrophone (Precision Acoustics LTD, DCPS 196). The attenuation was calculated using Equation (1):

(1) $$\alpha =\frac{1}{t\;\times T^{2}}\ln\frac{V_{0}}{V}$$

where α is the attenuation (dB/cm·MHz) of the warming biomaterial, t is the thickness of the membrane, T is the reflection coefficient, V_0_ is the initial voltage by hydrophone measurement, and V is the measured voltage of the warming biomaterial.

### 4.5. Ultrasonic Treatment

N2A cells were maintained in Dulbecco’s Modified Eagle Medium with 10% fetal bovine serum and 1% penicillin at 37 °C in a humidified 5% CO_2_ atmosphere. The subculture period was 3–4 days/week using trypsin, and medium change was done every 2 days. N2A cells were seeded on a 3.5 cm diameter dish and allowed to attach for 1 day in the incubator. The medium was then changed by adding 4 mL of fresh medium. The membrane was placed on the dish before ultrasonic treatment. The dish was put in a water-filled 6-well plate, then the ultrasonic probe was placed on the medium. The ultrasonic parameters used were 1.025 MHz and 52 mVpp. The treated group was divided into a negative control group (without membrane and ultrasonic treatment for 16 min), positive control group (without membrane and ultrasonic treatment for 43 min), and membrane group (with membrane and ultrasonic treatment for 16 or 43 min). N2A cells were continuously treated with ultrasound for 3 days, then cell proliferation, nerve outgrowth, and acetylcholinesterase (AChE) activity were analyzed.

### 4.6. Cell Survival Analysis

The cytotoxicity of membrane combined ultrasonic treatment was evaluated by WST-1 assay. The control and membrane groups were replaced by fresh medium containing 10% WST-1 solution and incubated for 3 h. Then 100 μL of medium from each group was pipetted into a 96-well plate and cell survival was measured through an ELISA reader.

### 4.7. Nerve Outgrowth Analysis

To investigate the efficacy of nerve outgrowth by membrane combined ultrasonic treatment, cytoskeleton staining was done, and nerve length was calculated. N2A cells were washed with phosphate-buffered saline (PBS) then fixed for 15 min with 3.7% formaldehyde. Cells were then washed with PBS and stained with tubulin antibody overnight. After washing the cells, Hoechst 33324 was used to stain the nuclei for 15 min. Cells were viewed under a fluorescence microscope. The length of nerve outgrowth from fluorescence images was quantified using MetaMorph software.

### 4.8. Analysis AChE Activity

To quantify the AChE activity of neuronal differentiation in membrane combined ultrasonic treatment, an acetylcholinesterase assay kit (Abcam) with RIPA Buffer was used in accordance with the manufacturer’s instructions. The concentration of proteins was measured using Bradford’s method with bovine serum albumin as the standard.

### 4.9. Statistical Analysis

The data are presented as mean ± SD. Significant differences between groups were identified by one-way or two-way analysis of variance followed by Tukey’s test as appropriate. *p*-values <0.05 were considered statistically significant.

## 5. Conclusions

In this study, magnetic nanoparticles (Fe_3_O_4_) were embedded into a PCL membrane to create a warming biomaterial to enhance neurite outgrowth on neural cells by ultrasonic treatment. The warming membrane had an excellent heating rate, which could shorten the exposure period of cells from the ultrasound. Through ultrasonic treatment, neurite outgrowth was induced, extending the axon length while also promoting AChE activity of neural cells. In future research, this warming biomaterial can be used as a nerve conduit for peripheral nerve injury therapy where the precise location of target tissues can be visualized. Moreover, the risk of overexposure can be avoided by reducing the treatment time.

## Figures and Tables

**Table 1:** Attenuation of warming biomaterial. PCL, polycaprolactone.

Sample	Attenuation (dB/cm·MHz)
Warming Biomaterial	739.99 ± 3.56
PCL	100.84 ± 0.87

## Abbreviations

FDA	Food and Drug Administration
CE	Conformit Europe
US	Ultrasound
AChE	Acetylcholinesterase
WM	Warming Membrane
DSC	Differential Scanning Calorimeters
MAPK	Mitogen-Activated Protein Kinase
PI3K	Phosphatidylinositol 3-Kinase
SEM	Scanning eEectron Microscope
PBS	Phosphate Buffered Saline
